# Adaptive control of human action: the role of outcome representations and reward signals

**DOI:** 10.3389/fpsyg.2013.00602

**Published:** 2013-09-09

**Authors:** Hans Marien, Henk Aarts, Ruud Custers

**Affiliations:** ^1^Department of Psychology, Utrecht UniversityUtrecht, Netherlands; ^2^Cognitive, Perceptual and Brain Sciences, University College LondonLondon, UK

**Keywords:** goal-directed action, motivation, adaptive control, outcome representation, reward signal

## Abstract

The present paper aims to advance the understanding of the control of human behavior by integrating two lines of literature that so far have led separate lives. First, one line of literature is concerned with the ideomotor principle of human behavior, according to which actions are represented in terms of their outcomes. The second line of literature mainly considers the role of reward signals in adaptive control. Here, we offer a combined perspective on how outcome representations and reward signals work together to modulate adaptive control processes. We propose that reward signals signify the value of outcome representations and facilitate the recruitment of control resources in situations where behavior needs to be maintained or adapted to attain the represented outcome. We discuss recent research demonstrating how adaptive control of goal-directed behavior may emerge when outcome representations are co-activated with positive reward signals.

## Introduction

Human goal-directed behavior is supported by a set of mental tools that tune action to dynamic environments. The question how this adaptive control process works has received a lot of attention in the literature (Morsella et al., 2009). Although there exist different conceptualizations, such as executive processes (Smith and Jonides, 1999), working memory operations (Baddeley, 2007), and cognitive control (Miller and Cohen, 2001), they share three basic components of control: active maintenance of goal-relevant information; inhibition of irrelevant information; and shifting of information (Miyake and Shah, 1999).

Most research on the control of human behavior considers the person as the agent of control (Locke and Latham, 1990; Bandura, 2001). People are assumed to control their behavior by setting goals, keeping them active in mind, and adapting their behavior when needed. More recent research adopts a mechanistic account by suggesting that adaptive control processes are self-emergent once a goal is activated (Braver and Cohen, 2000; Postle, 2006; Hazy et al., 2007). In line with this mechanistic account we take the activation of a goal as the starting point of our analysis, and address the question of how the self-emergent process may be modeled to understand how goals instantiate adaptive control.

Basically, two features are central to the control of goal-directed behavior. The first feature pertains to the notion that actions are represented in terms of outcomes. The second feature comprises the rewarding property or value of these represented outcomes. Research on ideomotor theory of action investigates the first feature by examining and explaining how action-effect knowledge is acquired and how outcome representations are implemented in action selection (Hommel, 2013). Research on the second feature investigates how rewarding or positive affective signals, such as positive mood (Aspinwall, 1998; van Wouwe et al., 2011), monetary gains (Muller et al., 2007; Heitz et al., 2008), or positively valenced outcome information (Custers and Aarts, 2005; Gable and Harmon-Jones, 2008) influence perception and cognition in action control.

In essence, both features work in tandem to control behavior adaptively. Whereas outcome representations serve as reference points for perception and action (Powers, 1973; Carver and Scheier, 1982), accompanying positive reward signals assign value or utility to outcomes (Shizgal, 1999) and facilitate the recruitment of executive control processes (Locke and Braver, 2010). However, a theoretical and empirical analysis of the combined role of these features has largely been neglected in the literature. Here, we aim to integrate research on the ideomotor principle and research on the role of reward signals in action control.

## The role of outcome representations in the control of behavior

Human goal-directed behavior is thought to result from the brain's capacity to predict and represent actions in terms of their outcomes (Suddendorf and Corballis, 2007). Activating an outcome representation prepares action in an offline fashion (i.e., planned ahead). However, engaging in goal-directed behavior requires knowledge about action-effect relationships. Action-effect learning has been extensively studied and provides an explanation for the emergence of outcome representations (Shin et al., 2010). Basically, a link between action and effect is formed when a consequence of a motor movement is observed and further strengthened if this effect occurs consistently. Because the link between action and effect is assumed to be bidirectional, this strengthened link can be used to produce a specific outcome. This is the ideomotor principle: activating an outcome representation readily selects the action (Hommel et al., 2001).

According to this principle, multiple outcome representations can be associated with multiple actions (Hommel, 1996; Kunde et al., 2002). This way, goal-directed behavior is structured around equifinality and multifinality sets. Multiple actions can thus serve one outcome or a single action can produce multiple outcomes, rendering goal-directed behavior adaptive (Kruglanski et al., 2002).

Initially the ideomotor principle explains action selection on a sensorimotor level. However, human behavior is more complex and involves goals that are further removed from direct motor activation. It can be suggested, though, that goal-directed behavior emerges from simple movement goals to complex social goals that are accessed in different contexts by the same mechanisms underlying action-effect learning (Maturana and Varela, 1987). We first learn to orchestrate our motor movements before we can effectively hit a light switch and illuminate a dark room. Eventually certain learned patterns of motor movements become associated with new observable outcomes in terms of sensory/perceptual and semantic/cognitive codes (Pulvermüller, 2005; Kray et al., 2006; Lindemann et al., 2006; Aarts and Veling, 2009). Indeed, it has been demonstrated that sensory-motor goal representations (acquired in goal-directed motor tasks) generalize to abstract features of outcomes, such that outcome representations can become socially meaningful (Beckers et al., 2002).

People rely on these outcome representations during action selection and execution. In cybernetic models of action control outcome representations serve as reference points (Adams, 1971). When an action produces an outcome not matching the pre-activated outcome representation, an action-related error signal is produced (Carter et al., 1998). Control is then necessary and should subsequently result in switching to a new course of action and inhibiting the old one. Active maintenance of the outcome representation thus often operates in concert with other adaptive control processes to attain the outcome.

## The role of reward signals in control

Ideomotor theorizing provides a parsimonious framework to understand how action-effect knowledge is acquired and how outcome representations are involved in the selection of action. However, it does not include specific predictions about when and how outcome representations gain control over behavior. There is a vast literature that does examine the emergence of adaptive control from an affective-motivational perspective.

First of all, there is research on the role of positive mood or emotion in cognitive control (Ashby et al., 1999; Fredrickson, 2004). This literature suggests that positive affect can broaden cognition (e.g., making people more creative) or funnel cognition (e.g., by focusing on local stimuli). Secondly, there is literature showing effects of prospective monetary gains on control processing such that effortful behavior can be boosted or strategically implemented (Bijleveld et al., 2012). Finally, the positive valence of outcome representations (acquired through evaluative conditioning procedures) can enhance effortful control in tasks generating the outcome (Custers and Aarts, 2010). These different lines of research suggest that positive affect, monetary gains and positive outcome representations serve as a general reward signal that acts as a common currency for modulating adaptive control (Shizgal and Conover, 1996), which either results in increased flexibility or more focused processing (Aston-Jones and Cohen, 2005). It remains unclear how the affective-motivational perspective deals with the question of when flexible or focused processing dominates. However, it is assumed that adaptive control processes originate from subcortical output releases of dopamine in the PFC, which is associated with the processing of general reward signals (Aarts et al., 2011; Chiew and Braver, 2011).

From this affective-motivational perspective, reward signals have been found to play a crucial role in each of the three basic components of adaptive control. Reward signals have been shown to (1) cause active maintenance of task relevant information and outcomes (Zedelius et al., 2011); (2) facilitate the inhibition of task-irrelevant information (Veling and Aarts, 2010); and (3) reduce switch costs in task-switching paradigms (Dreisbach and Goshke, 2004). These findings indicate the close relationship between adaptive control of human action and the processing of reward signals.

Reward-driven modulation of executive control is highly adaptive, because it justifies the allocation of limited cognitive resources (Pessoa, 2009). Resource allocation is guided by a principle of conservation such that effort will be expended only if it can be compensated by a significant benefit in the end (Brehm and Self, 1989; Gendolla et al., 2011). Reward signals thus ensure the recruitment of adaptive control processes when behavioral demands are imposed by environmental changes. Indeed, there are several studies that show how task demands and task incentives interact in producing effort intensity (Bijleveld et al., 2009; Silvestrini and Gendolla, 2013). In this research the conditions of demand are often explicitly communicated and it is shown that individuals invest effort only when the goal is attainable (i.e., moderately high demands) and valuable rewards are at stake. Thus, people seem to make trade-offs by weighing explicit information of reward value and demands. This raises the question of whether demand information needs to be explicit or whether such trade-offs also occur in contexts where differences in demands are less clear.

In a recent line of research we addressed this question using a modality shift paradigm (Marien et al., in preparation-a). Participants were instructed to respond to visual or auditory targets as fast as possible. Immediately before presentation of these targets we either presented a preparatory stimulus in the same modality as the target (ipsimodal trials, e.g., visual-visual), or a preparatory stimulus in a different modality (crossmodal trials, e.g., visual-auditory). The latter type of trials requires more resources (i.e., are more demanding) to respond to than the former type, because participants have to switch their prepared visual modality to the auditory modality. This typically results in a delayed response time caused by a modality switch cost, especially when this switch cannot be anticipated (Turatto et al., 2002). On half of the trials participants were presented with a 5 eurocents coin which they could earn; on other trials this reward signal of the coin was absent. Importantly, the preparatory stimuli were not predictive of whether a switch would occur or not. As expected, participants responded significantly faster when a reward was at stake during crossmodal trials, but there was no speeded responding during rewarded ipsimodal trials. Furthermore, the absence of the latter effect could not be explained by physical limits of speed of responding. Reward signals thus specifically reduce switch costs in an instrumental way, even in contexts that are ambiguous about task demands.

However, in most research on reward signals and cognitive control participants are instructed to perform a given action to obtain a specific outcome. Accordingly, research on the impact of reward signals on adaptive control is thus mainly limited to instructed task goals and does not consider how reward signals interact with outcome representations in controlling behavior (Dickinson and Balleine, 1994). We propose that analyzing the interplay between outcome representations and positive reward signals offers a more comprehensive examination of adaptive control of human action. In the next section, we discuss some recent research that examines this interplay in more detail.

## The combined role of outcome representations and reward signals

The combined role of outcome representations and reward signals has been examined to explore the building blocks of adaptive control in goal pursuit (Custers and Aarts, 2005, 2010). For instance, the activation of the outcome representation of physical exertion facilitated effortful control in action when this outcome representation was immediately followed by reward signals (i.e., positive words) in an evaluative conditioning procedure (Aarts et al., 2008). Participants resisted the pressure to release but persisted in squeezing a handgrip. Furthermore, this study provided evidence for the distinct roles of outcome representations and reward signals. The mere activation of the outcome representation facilitates initiation of the action, but did not increase control unless positive reward signals were attached to it. Several other studies have also demonstrated the function of reward signals in mobilizing action control (e.g., Capa et al., 2011; Köpetz et al., 2011; Veltkamp et al., 2011).

Building on this line of research, we investigated whether the pairing of positive reward signals with outcome representations translates into adaptive control in terms of making people more flexible in goal-directed behavior (Marien et al., 2012). In a modification of a set-switch paradigm (Dreisbach and Goschke, 2004), participants had to turn on a light by pressing either a left or a right key. On each trial, the correct response was indicated by a dot of a particular color appearing either left or right. A dot of a different color was presented in the opposite location, but had to be ignored. Before each trial, a cue appeared consistently reminding people of the outcome (turn on light). These cues were immediately followed by positive or neutral stimuli. After some trials, participants had to ignore the color they had to attend to earlier and react to a new color. Participants in the positive reward signal condition had significantly lower switch costs than those in the neutral condition. These findings suggest that being able to swiftly switch the course of action to obtain an outcome is dependent on whether the outcome representation of the action was co-activated with reward signals.

Whereas most studies on the combined role of outcome representations and reward signals in facilitating control consider the outcomes as given, from research on ideomotor theory one would expect that these outcome representations are normally acquired in daily life as a result of learning that the outcome follows from an action (Elsner and Hommel, 2001). Thus, according to our present analysis positive reward signals should only increase control when an action is represented in terms of its outcome. Specifically, only when the presentation of a specific stimulus follows an action rather than preceding it, will an accompanying positive reward signal cause people to engage in controlled behavior to obtain the outcome.

In a recent test of this idea (Marien et al., in preparation-b), participants had to execute an action (pressing a key) that was either preceded or followed by a stimulus on the computer-screen (e.g., the word “scissors”). The stimulus was accompanied by a neutral or positive reward signal by presenting a spoken word through headphones (e.g., the word “with” or “nice”). Thus, the stimulus represented an outcome of an action or not, and this outcome representation was co-activated with a reward signal or not. After some pairings, participants were presented with the stimulus on the screen and had to press another key repeatedly to move the stimulus closer to themselves in an easy way (one single key) or a more demanding way (multiple keys). Faster repetitive action in this task implies more control. Results showed that participants were faster in moving the stimulus to themselves only when it represented an outcome of their action and was co-activated with a positive reward signal. This effect was more pronounced when moving the stimulus to themselves was demanding. These findings suggest that adaptive control of goal-directed behavior is more likely to occur when positive reward signals accompany the process of representing action in terms of outcomes. Moreover, resources to control behavior seem to be allocated to obtain the outcome according to a principle of conservation (Silvestrini and Gendolla, 2013).

## Conclusion, implications, and prospects

We proposed that an integration of ideomotor accounts with affective-motivational accounts of action can shed new light on the control mechanisms underlying human goal-directed behavior. Although ideomotor theorizing offers a framework to understand how action-effect knowledge is acquired and how outcome representations select action, it is less explicit in predicting when and how control of behavior results from the activation of outcome representations. To understand the emergence of adaptive control reward signals should be taken into account. Although there is some research investigating the impact of reward signals on action-effect learning, the analysis is mainly focused on how it affects the binding strength and performance of the associated action (Muhle-Karbe and Krebs, 2012).

We also suggest that motivational accounts of adaptive control should incorporate more insights of ideomotor theory. Adaptive control processes are closely linked with reward processing, but the role of outcome representations is under-investigated in this literature. It is important for reward signals to connect with outcome representations in order for them to have a profound effect on adaptive control. The present analysis suggests that positive signals of different sources denote the value of an outcome and facilitate control of behavior. This implies that the influence of reward signals on recruiting executive control resources might not follow a direct path, but is mediated by the assigned value of the outcome representation. Future research could address (1) how personal value of an outcome representation results from reward signals, and (2) whether personal value mediates the instigation of control.

One way to approach this matter is by analyzing the neurocircuits prioritizing and controlling goals. Specifically, recent work in cognitive neuroscience proposes the involvement of specific neurotransmitter systems that cause people to exploit (being rigid to reach a goal) or to explore (prioritizing other goals) their environment (Aston-Jones and Cohen, 2005). Noradrenergic pathways in the brain are suggested to be associated with exploitation while dopaminergic pathways are supposed to be engaged in exploration.

This neurocircuit analysis of adaptive control can benefit from the present analysis. Adaptive control in terms of flexible or rigid/persistent processes may be dependent on the level of behavioral representation to which reward signals are attached. Goal-directed behavior is hierarchically structured (Botvinick, 2008), and hence the control of behavior may be directed at the level of action (means) representations or outcome (goal) representations depending on context and individual differences (Vallacher and Wegner, 1989). For example, goal-directed control of turning on a light may be identified and guided by the representation of “pressing the button” or “turning on the light.” So when representations of means are paired with reward signals action control is more likely to occur on the means level. Paradoxically, this could lead to more rigidity in control. We found that participants were less prone to switch to another action when the representation of the means was cued and paired with reward signals (Marien et al., 2012). In other words, when an outcome representation can be regarded as a subgoal of another outcome representation higher in the hierarchy (i.e., “pressing the key” in order to “turn on the light”), treating it with reward signals will increase local exploitive focus instead of broad explorative processing (Gable and Harmon-Jones, 2008). Taking the level of behavior representation into account may lead to specific predictions when reward signals produce a flexible or rigid mode of control.

Research on adaptive control of human action can advance by looking at outcome representations in combination with reward signals. It can especially help us to understand how the human mind functions optimally in the ever changing environment that we inhabit.

### Conflict of interest statement

The authors declare that the research was conducted in the absence of any commercial or financial relationships that could be construed as a potential conflict of interest.
